# The Severity of Autism Is Associated with Toxic Metal Body Burden and Red Blood Cell Glutathione Levels

**DOI:** 10.1155/2009/532640

**Published:** 2009-08-26

**Authors:** J. B. Adams, M. Baral, E. Geis, J. Mitchell, J. Ingram, A. Hensley, I. Zappia, S. Newmark, E. Gehn, R. A. Rubin, K. Mitchell, J. Bradstreet, J. M. El-Dahr

**Affiliations:** ^1^Division of Basic Medical Sciences, Southwest College of Naturopathic Medicine, Tempe, AZ 85282, USA; ^2^Department of Pediatric Medicine, Southwest College of Naturopathic Medicine, Tempe, AZ 85282, USA; ^3^Autism Research Institute, San Diego, CA 92116-2599, USA; ^4^Center for Integrative Pediatric Medicine, Tucson, AZ 85711, USA; ^5^Department of Mathematics, Whittier College, Whittier, CA 90601-4413, USA; ^6^International Child Development Resource Center, Phoenix, AZ, USA; ^7^Department of Pediatrics, Tulane University School of Medicine, New Orleans, LA 70112, USA

## Abstract

This study investigated the relationship of children's autism symptoms with their toxic metal body burden and red blood cell (RBC) glutathione levels. In children ages 3–8 years, the severity of autism was assessed using four tools: ADOS, PDD-BI, ATEC, and SAS. Toxic metal body burden was assessed by measuring urinary excretion of toxic metals, both before and after oral dimercaptosuccinic acid (DMSA). Multiple positive correlations were found between the severity of autism and the urinary excretion of toxic metals. Variations in the severity of autism measurements could be explained, in part, by regression analyses of urinary excretion of toxic metals before and after DMSA and the level of RBC glutathione (adjusted *R* ^2^ of 0.22–0.45, *P* < .005 in all cases). This study demonstrates a significant positive association between the severity of autism and the relative body burden of toxic metals.

## 1. Background

Autism is a severe developmental disorder which involves social withdrawal, communication deficits, and stereotypic/repetitive behaviour. The pathophysiological etiologies which precipitate autism symptoms remain elusive and controversial in many cases, but both genetic and environmental factors (and their interactions) have been implicated. One environmental factor that has received significant attention is the body burden of mercury, lead, and other toxic metals [1–5].

Bernard et al. [1] discussed the many similarities between the symptoms of children with autism and children poisoned by mercury. An epidemiology study by Windham et al. [2] found that the amount of airborne pollutants, and especially mercury, correlated with an increased risk for autism. A study by DeSoto and Hitlan [3] found that blood levels of mercury did significantly correlate with the diagnosis of autism. A small study by Adams et al. [4] found that children with autism had a 2-time higher level of mercury in their baby teeth than typical children. A study by Bradstreet et al. [5] investigated the body burden of toxic metals by giving dimercaptosuccinic acid (DMSA), an oral chelation medication approved by the FDA for treating infantile lead poisoning. They found that the children with autism excreted 3.1 times as much mercury into their urine (which is where DMSA is excreted), *P* < .0002, but lead and cadmium levels were not significantly different. Overall there is some evidence to suggest that mercury and possibly other toxic metals are related to the etiology of autism.

This study investigates the possible relationship of the severity of autism to the relative body burden of toxic metals. The severity of autism was assessed using four tools, a professional evaluation based on the Autism Diagnostic Observation Schedule [6], and parental evaluations based on the Pervasive Developmental Disorders Behaviour Inventory (PDD-BI) [7], the Autism Treatment Evaluation Checklist (ATEC) [8], and the Severity of Autism Scale (SAS). The individual burden of toxic metals was assessed based on urinary excretion, both before and after taking oral dimercaptosuccinic acid (DMSA). DMSA is a licensed medication for treating lead poisoning and indicated in cases meeting toxic criteria. DMSA is, however, widely used off-label for other metal exposures, for example, mercury. It acts by forming sulfhydryl linkages to divalent metal cations, forming a chelated metal complex which is then excreted in the urine [9]. Urine measurements before and after taking DMSA provide an indication of both ongoing environmental exposures (before DMSA provocation) and the accumulated or relative body burden (postprovocation with DMSA). Red blood cell (RBC) glutathione was measured because it is one of the body's primary means for excretion of toxic metals.

This paper investigates the possible relationship of the severity of autism to the body burden of toxic metals and RBC glutathione levels. This paper is part of a larger study which investigates the safety and efficacy of DMSA therapy, including both the biological consequences [10] and the DMSA associated behavioural effects [11]. The larger study involves a 3-day round of DMSA, to screen for admission into a 3-month DMSA treatment study; only children with high levels of urinary toxic metals were admitted into the long-term 3-month treatment study. 

## 2. Methods

The methodology is discussed in detail in the companion paper [10]. Briefly, this study was conducted with the approval of the Human Subjects Institutional Review Board of Southwest College of Naturopathic Medicine. All parents and (where possible) children signed informed consent/assent forms. The study participants were recruited in Arizona, with the help of the Autism Society of America—Greater Phoenix Chapter and the Arizona Division of Developmental Disabilities. 

The entry criteria were the following.

1. Children with autism spectrum disorder, diagnosed by a psychiatrist, psychologist, or developmental pediatrician.
2. Age 3–8 years.
3. No mercury amalgam dental fillings (due to a concern of their interaction with DMSA).
4. No previous use of DMSA or other prescription chelators.
5. No anemia or currently being treated for anemia due to low iron.
6. No known allergies to DMSA.
7. No liver or kidney disease.
8. Children are well hydrated (receiving adequate daily intake of water).

Four metrics were employed to assess the severity of autism: the PDD-BI, ATEC, SAS, and ADOS. Multiple assessment instruments were selected because they each provide insights into various aspects of autism. The ATEC was completed approximately 2-3 weeks prior to taking the DMSA, and the other three instruments were completed approximately 2–4 weeks after the initial 3-day round of DMSA, for children whose excretion of toxic metals was deemed high enough to warrant continuation in the long-term treatment study. The ATEC, PDD-BI, and SAS were assessed by the participant's parents, and the ADOS evaluation was performed by a certified ADOS evaluator. It should be noted that the ADOS was developed primarily for diagnosing autism, whereas the other tools were developed for assessing changes in autistic symptoms during treatment studies. 

DMSA was administered orally in 9 doses of 10 mg/kg, 3 times daily, over 3 days. Urine was collected for approximately 8 hours prior to taking the DMSA, and for approximately 8 hours immediately after the 9th dose, in a process similar to a previous retrospective study of relative body burden of heavy metals [2]. RBC glutathione was measured approximately 1-2 weeks prior to taking the DMSA. The details of measuring the urinary metals and RBC glutathione are given in [10].

The PDD-BI is composed of many subscales. One of the subscales, the Semantic/Pragmatic Problems (SPPs), was difficult to interpret, since children with no spoken language inappropriately scored as less severely affected than those with limited language. Therefore, we exclude the SPP subscale in the Autism Composite score, resulting in a modified Autism Composite score consisting of Sensory/Perceptual Approach, Ritualisms/Resistance to Change, Social Pragmatic Problems, Social Approach Behaviors, Phonological and Semantic Pragmatic subscales. This modified Autism Composite score was discussed with I. Cohen, the developer of the PDD-BI. We believe that this modified subscale is more useful because several children initially without speech began talking after DMSA treatment in the study. The development of speech led to a worsening of their score on the SPP, because a nonverbal child is given a score of zero (indicating no semantic/pragmatic problems, which is the same score a typically developed child would receive) compared to a child with limited speech but major semantic/pragmatic problems who would receive a high score on the SPP. Thus, we think the modified Autism subscale (without the SPP) is more useful for children with very limited or no language.

In order to assess global changes in autism severity, a new metric was developed for this study. The Severity of Autism Scale (SAS) is introduced for the first time in this series of papers. It is essentially a Clinical Global Impression scale using a 0–10 severity scale, with the difference being that the scale was made specific to autism by defining the numeric values (see below). The purpose of the tool is to provide a simple, overall assessment of the severity of the symptoms of autism. In this study we will analyze the correlation of this scale with the other more established assessment tools.

**Severity of Autism Scale:**

0: normal,
1: slight symptoms of autism,
2–4, mild symptoms of autism,
5–7, moderate symptoms of autism,
8–10, severe symptoms of autism.

63 participants were assessed with the ATEC, and 49 participants were assessed with the PDD-BI, SAS, and ADOS. Fewer participants were assessed for the latter three tests because some participants had low urinary excretion of toxic metals and were not eligible to continue, and some participants dropped out. 

Table 1 lists the characteristics of the participants. 

Table 2 lists their average urinary excretion of toxic metals before and after taking DMSA.

### 2.1. Regression Analysis

Regression analysis was employed to examine the relationship between the severity of autism (assessed by the ATEC, PDD-BI, SAS, and ADOS) and the urinary excretion of toxic metals, (both before and after taking DMSA), and further with the initial glutathione (in the red blood cells). For the selected dependent and independent variables, stepwise linear regression analyses were conducted: initially all independent variables were included in the regression; then at each step, the variable with the highest *P*-value was eliminated, and this process was continued until the adjusted *R* ^2^ value began declining. Thus, the goal was to determine the best fit to the sample data for the selected model, taking into account the correlation among the independent variables. Since the data had several missing values (due to missing lab or behavioural data), the regression analyses were conducted in two slightly different ways which generally yielded very similar results: ( 1) eliminate all participants with missing data for any of the variables in the model at the beginning of the analysis, and ( 2) eliminate participants on an as-needed basis (i.e., only where there is missing data for any variable in the current step in the analysis). Since these two methods yielded very similar results, for brevity we only report the results for method 1.

## 3. Results

### 3.1. Correlations of Severity Scales

Table 3 shows the correlations among the assessment scales. There is a high correlation between the ATEC and the PDD-BI (*r* = 0.87), and a good correlation of the SAS with the ATEC (*r* = 0.70) and the PDD-BI (*r* = 0.72). The correlation of the ADOS with the other scales is somewhat lower (*r* = 0.60–0.67), probably since the ADOS evaluation was done by a professional evaluator, whereas the other assessments were done by the same parent.

### 3.2. Correlation Analysis

Table 4 shows the results of a simple correlation analysis of severity of autism versus toxic metal levels. Correlations with a *P*-value of less than .05 are shown in bold. Baseline excretion of antimony (Sb) and excretion of lead (Pb) after the 9th dose of DMSA are the two most consistent factors, although other metals also have *P* < .05 for some of the severity scales. In all cases for *P* < .05, the correlations are positive, so that high levels of toxic metals correlate with higher severity of autism, as expected. Also, the initial glutathione correlates positively with two of the severity scales at *P* < .05.

However, because we are analyzing many correlations, a traditional *P*-value of < .05 is not a rigorous guide. Since we are analyzing 76 possible correlations, random chance alone would result in approximately 4 results at *P* < .05. We found 13 instances of *P* < .05 for toxic metals, and the probability of that occurring randomly is 7 × 10^−5^, so it is very likely that most, but probably not all, of the correlations represent actual relationships. 

One way to deal with the problem of multiple correlation analyses is the Bonferroni approach. Using this approach involves dividing the nominal *P*-value by the number of tests, so that for 95% confidence one needs a *P*-value less than .05/76, or *P* < .0007. Using the Bonferroni approach, the correlations between initial Severity of Autism Scale (SAS) and baseline excretion of lead (Pb) and antimony (Sb) are significantly different from 0 at the 95% confidence level, and these are the only pairs that meet the Bonferroni criterion for the 5% significance threshold. Again, it should be noted that this is a conservative approach, designed to ensure that very few nonsignificant correlations are misrepresented as significant.

False discovery rate (FDR) is a less conservative method for performing multiple hypothesis tests, based on controlling the expected number of false positives among the cases declared significant. If we use FDR on the summary severity scores, then in addition to the results obtained from the Bonferroni analysis, the correlation between Initial ATEC Total and baseline excretion of antimony (Sb), and the correlations between Initial PDD-BI Autism Total and baseline excretion of antimony (Sb) and 9th dose excretion of lead (Pb) are significantly different from 0; we will term these findings “marginally significant.”

Next, consider the numbers of positive and negative sample correlation coefficients in the table. If there were no statistically significant correlations between autism severity and biological measures then we would expect on average about equal numbers of positive and negative sample correlation coefficients. For the summary severity measures, we observed 63 positive sample correlation coefficients (*r*'s) and 13 negative *r*'s. This corresponds to a *P*-value of 3 × 10^−9^ for the hypothesis that there is no correlation between the severity measures and biological measures. Thus it is extremely likely that there is a high overall positive correlation between the severity measures as a group and the biological measures taken as a group. 

Finally, the average of all of the 76 sample correlation coefficients is 0.14. If there were no statistically significant correlations between autism severity and the biological measures, the average of 76 sample correlation coefficients (each of which was taken form a sample of size 40 or more) would come from a distribution with mean 0 and standard deviation equal to 0.02. Under those conditions, the *P*-value for a mean correlation coefficient of 0.14 is less that 10^−10^, so again it is extremely likely that there is a high overall positive correlation between the severity measures as a group and the biological measures taken as a group.

Since multiple correlations were obtained, it was decided to conduct regression analyses, which are discussed in the next section. Basically, a regression analysis allows for the simultaneous consideration of multiple factors, such as how well certain combinations of different toxic metal excretions can predict values of a specific autism severity measure.

### 3.3. Regression Analyses of Initial Severity of Autism

Table 5 shows the results of stepwise linear regression analyses for the various autism severity scales as a function of urinary excretion of toxic metals (at baseline and after the 9th dose of DMSA) and initial glutathione. All of the analyses found that the variations in the severity of autism could be partially explained by the urinary excretion of toxic metals and initial glutathione, with adjusted *R* ^2^ values ranging from 0.22 to 0.45, and *P*-values all below .005. For the ADOS (which had the highest adjusted *R* ^2^), the most significant variables were mercury (Hg) and antimony (Sb) at baseline and mercury and tungsten (W) at the 9th dose.

Since the ADOS score had the highest adjusted *R* ^2^ values, we also conducted a similar regression analysis on the subscales—(a) language and communication; (b) reciprocal social interaction; (c) play; (d) stereotyped behaviors and restricted interests (SBRIs). Those results are show in Table 6. The variation in all four of the ADOS subscales could also be partially explained by urinary excretion of toxic metals and RBC glutathione (adjusted *R* ^2^ of 0.21–0.41, *P* < .02 in all cases). The ADOS Sociability and the ADOS Communication subscales had the highest adjusted *R* ^2^ (0.41 and 0.37, resp.). For the ADOS Sociability subscale, the most significant variable was tungsten at the 9th dose, followed by tungsten, aluminum, and thallium at baseline and lead and thallium at the 9th dose. For the ADOS Communication subscale, the most significant variables were mercury (at baseline and 9th dose) and antimony (Sb) at baseline. 

Since the toxic metal excretions exhibit considerable correlation amongst themselves [10], one should refrain from reading too much into the relationships between specific metals and severity of autism and instead should interpret the results as indicating a general relationship between autism severity and urinary excretion of toxic metals. 

## 4. Discussion

The different assessment tools were found to be highly correlated, which generally supports the validity of each of the assessment tools. The correlations were the highest between the modified PDD-BI and the ATEC, suggesting that those scales are very consistent. The ADOS had a lower correlation with the other scales; this at least partly due to different evaluator for the ADOS (assessed by a professional certified in the ADOS) versus the ATEC, modified PDD-BI, and SAS which were assessed by the same person (the parent who was the primary care giver). 

The various correlation analyses found that overall there were multiple positive correlations between the severity of autism and the urinary excretion of some toxic metals (both before and after taking DMSA). Lead (after DMSA) and antimony (at baseline) had the most consistent effect, but other metals were also important. The existence of multiple positive correlations suggested that a regression analysis was appropriate.

The regression analysis found that the body burden of toxic metals (as assessed by urinary excretion before and after DMSA) was significantly related to the variations in the severity of autism, for each of the four scales. The metals of greatest influence were lead (Pb), antimony (Sb), mercury (Hg), tin (Sn), and aluminum (Al). Different metals are significant for the different scales, and this partial disagreement is probably due to two factors. First, the severity scales are not identical, having somewhat different questions and evaluating symptoms somewhat differently; as pointed out in Table 3, the correlations between the scales are good but not identical. Second, it should be noted that the high correlation between urinary excretion of many of the metals (see Adams et al. [10]) makes it difficult to separate the effect of one metal from another. This makes it improper to assign too much meaning to specific regression variables and their coefficients. Thus, it is probably best to not overinterpret the results in terms of a particular metal, but to instead interpret them as evidence of the general role of toxic metals in relation to the severity of autism. Since oxidative stress and thiol metabolic disturbances have both been described in the autism population [12, 13], it is likely that these play a role in both relative burden and susceptibility to heavy metals. And since heavy metal exposure generates oxidative stress and thiol depletion, the potential etiological role of metal cations in generating autism symptoms should be further studied. Similarly, prior depletion of thiols and increased oxidative stress makes it more likely the individual will accumulate metals. 

It should also be noted that each severity scale assesses a somewhat different aspect of autism; for example, the ATEC has a major section on physical health, which is not assessed by the other scales. So, that may also explain why the different scales have somewhat different relationships with different metals. 

The ADOS had the highest adjusted *R* ^2^ value, suggesting that it is a very useful scale for assessing the severity of autism and for inclusion in correlation and regression analyses with biological factors. This may be due to the fact that, of the four tools we used, only the ADOS involves a trained professional making a quantitative assessment of many children, whereas the other tools are assessments by parents of only their child.

The strong correlation of the SAS with the other scales, and the high adjusted *R* ^2^ value (0.36), suggests that the SAS is a useful tool for simple assessment of the severity of autism.

We are aware of two other studies which found a relationship between the severity of autism and a biomarker related to heavy metal toxicity. One study by Geier et al. [14] found that elevations in urinary porphyrins (associated with mercury or lead and mercury toxicity) were significantly associated with Childhood Autism Rating (CARS) scores. A second paper to report a relationship of the severity of autism with a biomarker was a study which found a strong inverse relationship of the severity of autism with the amount of mercury in the baby hair of the subjects [15]. However, a replication study [16] did not reproduce that correlation with severity. So, while two studies [14, 15] do support a possible relationship of variations in the severity of autism with body burden of toxic metals, as was found in this paper, additional research is needed to confirm this finding.

This paper has focused on the possible relationship between toxic metals and the severity of autism. It has not included an examination of the source of those metals. Mercury, lead, and other toxic metals come from many sources. There has been particular interest in the possible relationship of autism and thimerosal (a mercury-based preservative once used in many childhood vaccines, but removed from most vaccines after 2003). However, this study was not designed to determine the sources of the toxic metals found in children with autism.

### 4.1. Limitations of this Study

The original study was designed primarily for evaluating the safety and efficacy of DMSA therapy. It was not primarily designed for investigating the relationship of the severity of autism to toxic metals, but that was an interesting outcome, so we felt it worthwhile to report it. Some limitations of the study design include the following.

1. The PDD-BI, SAS, and ADOS were assessed 2–4 weeks after the first round of DMSA, whereas the ATEC was assessed before. However, the strong correlation of the ATEC and PDD-BI suggests that this was a minor issue, and that the initial round of DMSA did not significantly affect the assessment.
2. The ATEC involved the largest number of participants (*n* = 63), whereas the other assessments involved somewhat smaller numbers (*n* = 49).

## 5. Conclusions

Overall, the correlation analysis found multiple significant correlations of severity of autism and the urinary excretion of toxic metals, such that a higher body burden of toxic metals was associated with more severe autistic symptoms. The results of the regression analyses (*P* < .005 in all cases) indicate that variations in the severity of autism may be partially explained in terms of toxic metal body burden. However, the finding of a relationship does not establish causality.

## Figures and Tables

**Table 1:** Characteristics of participants. The second number is the standard deviation.

	Total
Total participants	63
Male	57
Female	6
Age (years)	5.6 ± 1.6
Diagnosis	62 autism, 1 Asperger's

ATEC (total)	62 ± 28
SAS	5.1 ± 2.2
ADOS (communication + social)	15.8 ± 6.5
PDD-BI (modified autism score)	−54.3 ± 62

RBC glutathione (pre-DMSA)	501 ± 246 micromolar

**Table 2:** Urinary excretion of toxic metals in Phase 1, at baseline and after 9th dose of DMSA, in mcg/g creatinine. Creatinine values have units of mg/dL. *N* = 63. The metals are listed in approximate order of effect of DMSA on excretion. Significant results are highlighted in bold font.

Element	Baseline	After 9th dose	After 9th dose versus baseline
Pb	1.3 ± 2.3	9.2 ± 7.8	638%****
Sn	2.3 ± 3.4	9.7 ± 24	314%**
Bi	0.18 ± 0.45	0.41 ± 1.0	128%**
U	0.015 ± .04	0.031 ± .1	111%
Hg	0.86 ± .92	0.97 ± 0.88	13%
Tl	0.15 ± 0.12	0.21 ± 0.19	42%***
Sb	0.10 ± 0.10	0.14 ± 0.20	42%*
W	0.3 ± 0.29	0.46 ± 0.50	18%**
Al	16 ± 21	19 ± 33	21%
Ni	6.7 ± 5.1	7.6 ± 4.3	12%
Cd	0.38 ± 0.24	0.3 ± 0.23	−14%
As	32 ± 20	25 ± 18	−19%**
Creatinine	94 ± 52	80 ± 43	−15%**

*P < .1, **P < .05, ***P < .01, ****P < .001

**Table 3:** Correlation of autism severity scores.

	ATEC total	SAS	ADOS (social + communication)	PDD-BI (modified autism score)
ATEC	1			
SAS	0.70	1		
ADOS	0.60	0.60	1	
PDD-BI	0.87	0.72	0.67	1

**Table 4:** Correlation analyses of initial autism severity versus urinary metal excretion and initial glutathione. The metal excretion is measured both at Baseline (before DMSA) and after the 9th dose of DMSA. The first number in each cell is the correlation coefficient (*r*) and the second number is the *P*-value. Correlation coefficients with *P* < .05 are in bold. The last 2 rows list the total number of positive and negative correlation coefficients, respectively.

	ATEC total	ADOS (social + communication)	SAS	PDD-BI (modified autism score)
PbBase	−0.00 (0.96)	0.11 (0.47)	**0.50 (0.0002)**	0.22 (0.12)
SnBase	0.16 (0.32)	−0.11 (0.47)	0.12 (0.41)	0.09 (0.52)
TlBase	0.13 (0.32)	0.10 (0.49)	0.21 (0.15)	0.25 (0.077)
HgBase	0.13 (0.33)	0.05 (0.76)	0.15 (0.31)	0.18 (0.19)
SbBase	**0.40 (0.002)**	**0.35 (0.02)**	**0.51 (0.0002)**	**0.42 (0.0023)**
Wbase	0.16 (0.23)	0.07 (0.67)	0.26 (0.07)	0.17 (0.22)
AsBase	0.04 (0.74)	−0.05 (0.73)	0.00 (0.98)	0.04 (0.76)
CdBase	0.00 (1.00)	0.11 (0.48)	0.03 (0.83)	−0.10 (0.48)
AlBase	−0.06 (0.65)	−0.19 (0.20)	−0.05 (0.73)	0.02 (0.87)
Pb9	**0.27 (0.04)**	**0.34 (0.02)**	**0.36 (0.01)**	**0.42 (0.0027)**
Sn9	−0.02 (0.88)	0.00 (0.98)	0.02 (0.87)	−0.12 (0.42)
Tl9	**0.26 (0.046)**	0.11 (0.51)	0.27 (0.064)	0.24 (0.098)
Hg9	0.09 (0.52)	0.20 (0.18)	−0.02 (0.91)	0.07 (0.59)
Sb9	0.03 (0.84)	0.20 (0.19)	**0.38 (0.008)**	0.26 (0.065)
W9	0.11 (0.42)	**0.34 (0.02)**	−0.00 (0.99)	0.19 (0.18)
As9	0.19 (0.16)	−0.24 (0.12)	−0.24 (0.11)	−0.04 (0.79)
Cd9	0.07 (0.58)	**0.34 (0.024)**	0.08 (0.57)	0.15 (0.29)
Al9	0.06 (0.65)	0.28 (0.059)	0.25 (0.089)	0.17 (0.24)
Glut1	**0.25 (0.04)**	**0.34 (0.024)**	0.25 (0.09)	0.26 (0.70)
Number of positive coefficients	17	15	15	16
Number of negative coefficients	2	4	4	3

**Table 5:** Regression analyses of initial autism severity versus urinary metal excretion and initial glutathione. In the regression equation, the suffixes for the metals refer to the value at Baseline (B) and after the 9th ( 9) dose of DMSA in Phase 1.

	Adjusted *R* ^2^	*P*-value	Equation	Most significant variables
ATEC	0.22	.003	24.1–6.17 HgB + 76.6 SbB + 0.593 Pb9 + 3.97 Hg9 + 0.27 As9	SbB**, Pb9*
SAS	0.36	.002	4.81 + 1.70 PbB + 4.87 TlB − 0.640 HgB + 5.48 SbB − 1.87 CdB − 0.0237 AlB − 0.114 Pb9 − 3.14 Tl9 + 6.07 Sb9	PbB**
ADOS (comm. + social)	0.45	.0003	13.19–4.29 HgB + 24.1 SbB − 3.67 WB − 0.0673 AlB + 2.75 Hg9 + 6.60 W9 − 0.0539 As9 + 0.0054 Glut	HgB**, SbB*, Hg9*, W9*
PDD-BI (modified autism score)	0.25	.004	−131.8 + 70.4 WB − 0.789 Sn9 + 18.8 Hg9 + 255 Sb9 + 21.8 W9	Sb9**, WB*, Sn9*

**P < .01, *P < .05

**Table 6:** Regression analyses of initial ados subscales versus urinary metal excretion and initial glutathione. In the regression equation, the suffixes for the metals refer to the value at Baseline (B) and after the 9th ( 9) dose of DMSA in Phase 1.

	Adjusted *R* ^2^	*P*-value	Equation	Most significant variables
ADOS-Sociability	0.41	0.004	8.70 + 1.20 PbB + 0.217 SnB + 12.7 TlB − 1.64 HgB − 10.2 SbB − 2.61 CdB − 0.631 AlB − 0.186 Pb9 − 7.13 Tl9 + 6.27 Sb9 +6.15 W9 +3.62 Cd9	W9***, AlB**, TlB*, HgB*, Pb9*, Tl9*
ADOS-Commun.	0.37	0.0003	2.39–2.57 HgB + 23.1 SbB + 2.32 Hg9 + .0048 Glut	HgB**, Hg9**, SbB**
ADOS-Play	0.24	0.004	1.20 + 0.540 PbB + 4.23 Sb9 + 0.0017 Glut	PbB*
ADOS-SBRI	0.21	0.02	4.64–0.897 HgB − 2.63 CdB − 0.26 AlB + 0.050 Pb9 + 0.730 Hg9	

***P < .001, **P < .01, *P < .05
